# Cross-sectional study about the activities for various occupational groups on board during different voyage stages

**DOI:** 10.1186/s12995-019-0233-1

**Published:** 2019-05-03

**Authors:** Marcus Oldenburg, Hans-Joachim Jensen

**Affiliations:** Institute for Occupational and Maritime Medicine Hamburg (ZfAM), University Medical Center Hamburg-Eppendorf (UKE), Seewartenstrasse 10, 20459 Hamburg, Germany

**Keywords:** Maritime, Vessel, Activity profile, Phase of the voyage, Occupational groups

## Abstract

**Background:**

Employees on board must be highly qualified in order to be able to independently meet the different work requirements during the three voyage stages of a ship (port stay, river and sea passage). In this study, the activity profiles of the various occupational groups on container ships are presented according to the voyage stages.

**Methods:**

As part of a maritime field study on 22 container ships in the North Sea area, the work processes of four different professional groups on board were evaluated, and a list of activity profiles was compiled. Directly after a voyage stage, the 323 seafarers participating in the study recorded the duration of each task within the recent voyage stage. The average proportion for each activity was determined and presented as a job activity profile.

**Results:**

According to this profile, the diversity of tasks for the nautical officers and the deck ratings differ between the voyage stages. For watch officers, the focus of activity during port stay is on the preparation and monitoring of the loading process. During river and sea passages, more than 50% of the working time consists of monitoring the navigation area and about 10% of navigation. The main tasks for deck ratings during port stay include (preparation and follow-up) activities for loading and unloading the vessel and, during the other voyage stages, cleaning, painting and maintenance work on the ship. The activity profile for technical officers and engine room ratings less often differs significantly between the various voyage stages. There are numerous control, repair and maintenance tasks during the entire voyage.

**Conclusions:**

The established activity profiles show that the work diversity, especially among nautical officers and deck ratings, differs with a variety of requirements between the voyage stages. The activities of all four occupational groups varied most during port stay and less during the sea passage. To prepare maritime trainees for the expected job-related requirements and to identify the most suitable opportunities for recreation during a voyage, future maritime studies about stress on board should take the differences in the activities between occupational groups and the voyage stage into account.

## Background

The crew of a merchant ship is on its own on the high seas. Therefore, all necessary activities, be it those involved in maintaining the ship, in loading operations (e.g. fuel bunkering, loading and unloading, administrative tasks) or in providing for the crew on board (e.g. food supply and preparation, functionality of sanitary facilities, medical care) have to be carried out independently by the ship’s crew [1, 2].

Container ships are usually equipped with their own workshop so that the necessary tools and materials for regular maintenance and repair work on board are available. The large dimension of the ship’s operating system, the loading and transport of sometimes several thousand ship containers, the physical effects of the ship’s movements and last but not least the permanent influence of corroding salt water are a great burden on the ship. To reduce wear and tear and thus premature aging of the ship, the effort for maintenance and repair work on board should not be underestimated [3].

In addition to these routine tasks, unpredictable job demands often arise on board (for example, in the event of technical equipment failure). In the case of major technical damage, qualified specialists on land can be called in at the nearest port, if necessary. The next, often foreign port is sometimes only reached after several days at sea and there is no certainty that the necessary know-how or the required replacement material are available there. Any deviation from the planned course of a ship is associated with significant operating costs [4]. A longer stay in port due to land-based repairs also means high costs; therefore, shipping companies expect that regular routine repairs as well as maintenance and necessary acute repairs are carried out on the high seas by the crew themselves. This requires an appropriate qualification of the seafarers and is reflected in the job descriptions for the employees on board [1]. In total, it is necessary that the shipboard crew independently carries out a wide variety of different tasks on board.

Due to long-term separation from their families permanent influences of physical effects on the high seas (e.g. noise, vibration, ship movements, and heat) and long working hours, seafarers normally experience a high stress level during their shipboard stay over several months at a stretch [5–7]. Several studies have described these extraordinarily challenging working and living conditions as a possible cause of fatigue, psychophysical exhaustion or even health impairment [8, 9]. It is assumed that the activity profile of the shipboard crews may also result in high psychophysical stress.

To date, there is no percentage-weighted analysis as quantitative measure available about the activities for crews aboard container ships as the most prominent vessel type in merchant shipping [10]. In particular, no differentiation has been made so far between different phases of the voyage (port stay, canal and sea passage). The activity profiles for ships’ crews, however, differ between these three voyage stages [11]. Therefore, this study presents the activity profiles for the various occupational groups on container ships, taking the vessels’ voyage stage into account. The main purpose of the present study is to assess the seafarers’ activity profiles, which may indicate the opportunities for prevention. This may serve to improve the preparation of the crews in respect of the expected job-related requirements. Maritime trainees can obtain a more precise insight into the diversity of activities for the various occupational groups. In further studies, these findings may also support efforts to improve the work organization aboard.

## Methods

An examiner with experience in seafaring accompanied 22 sea voyages on German container ships (16 small feeder container ships in exclusive coastal travel and 6 large container ships with current deployment in the coastal area). During the voyages, 323 seafarers were asked about their typical activity profile on board (participation rate 88.5%). More than 95% of German shipping companies had previously been contacted. Out of these shipping companies, 12 were randomly selected and considered to be representative (in terms of crew structure and underlying safety standards) of the German fleet of container ships with current deployment in the coastal area.

The study sample consisted of 67 nautical officers, 158 deck ratings, 55 technical officers and 43 engine room ratings. The average age of the exclusively male crew members was 38.2 years (± 11.7 years). Participation in the study was completely voluntary and the data collected was anonymous. All participants gave their written informed consent before taking part in this study. The study was approved by the Ethics Committee of the Hamburg Medical Association (no PV4395).

Two maritime psychologists had conducted several standardized interviews with seafarers (nautical and technical officers and ratings), shipping companies (nautical and technical supervisor) and maritime stakeholders about the activities of the different occupational shipboard groups. Beside this information, several maritime job-descriptions have been used to develop an activity profile list for the different occupational groups during the three voyage stages. At the end of a typical voyage stage, the seafarers taking part in this study recorded the proportion of working time spent on tasks with each of the listed activities in the protocol. They also had the opportunity to enter additional activities in this list that had been relevant during their recent voyage stage.

### Statistical analysis

**Fig. 1:**
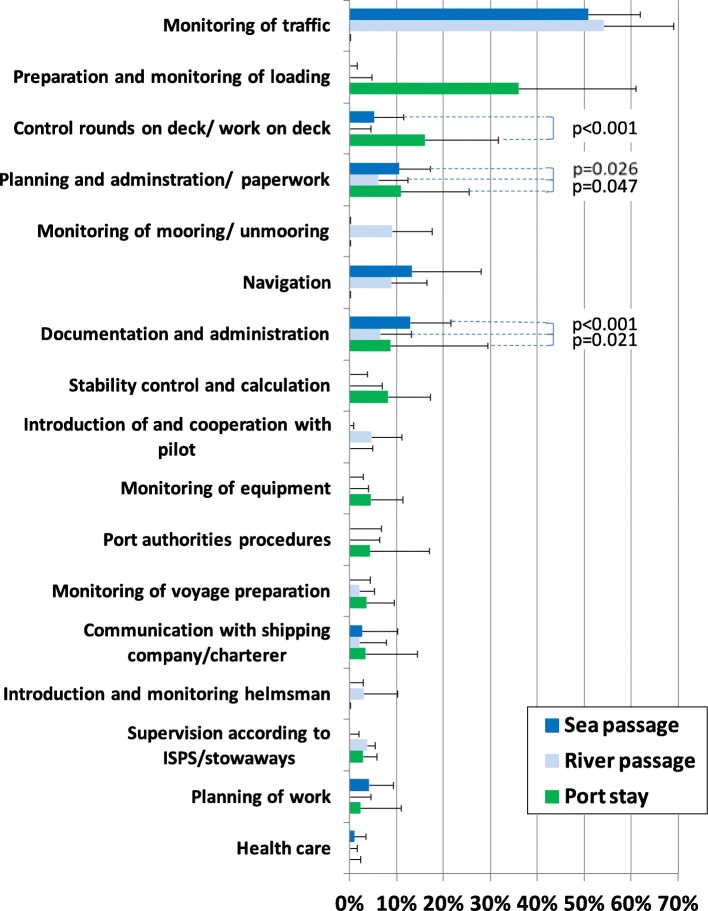
Activity profile for nautical officers. ISPS = International Ship and Port Facility Security Code. Mann-Whitney U test. p test only in cases of significant differences between the voyage stages (each higher than 0%)

**Fig. 2:**
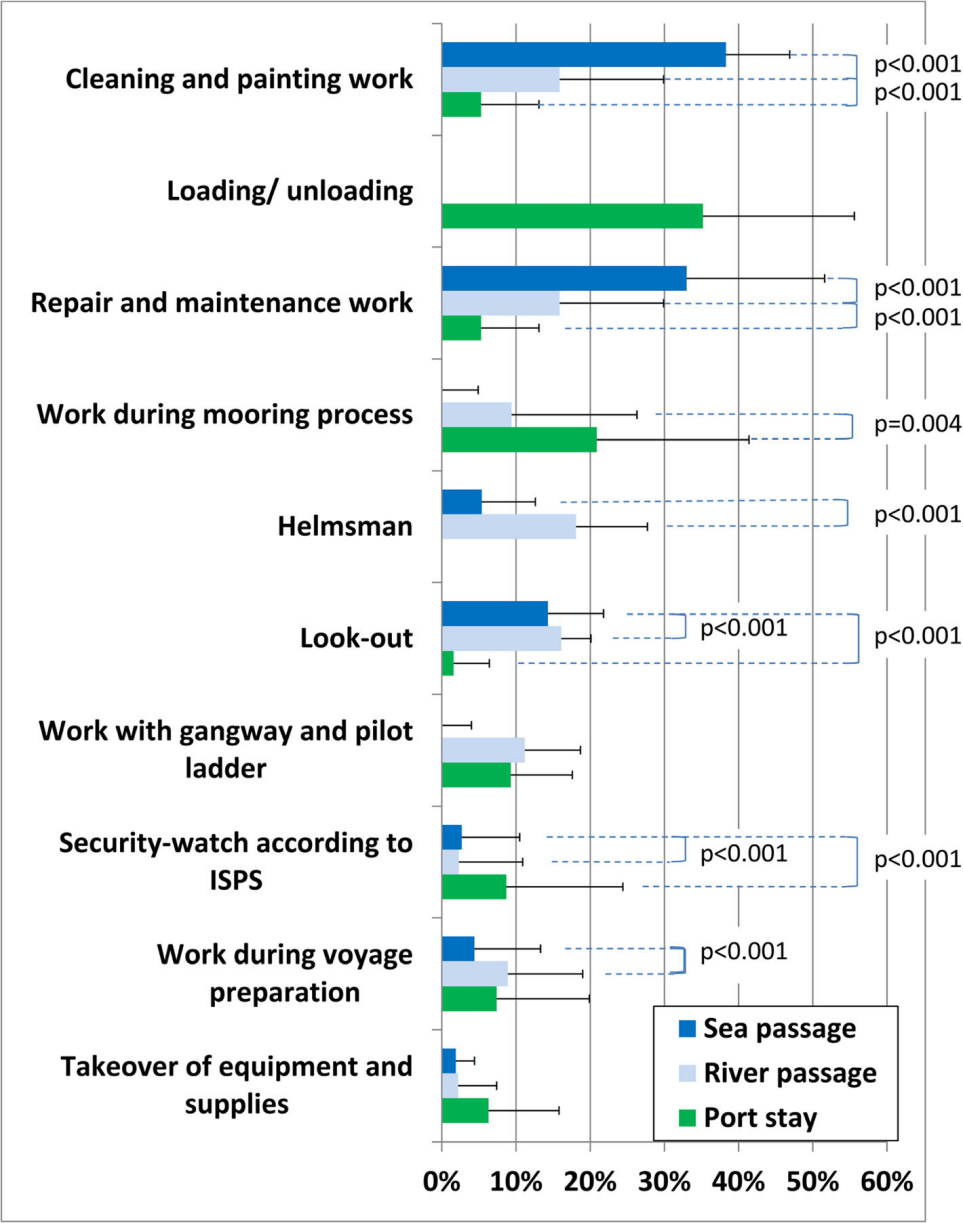
Activity profile for deck ratings. p test only in cases of significant differences between the voyage stages (each higher than 0%)

**Fig. 3:**
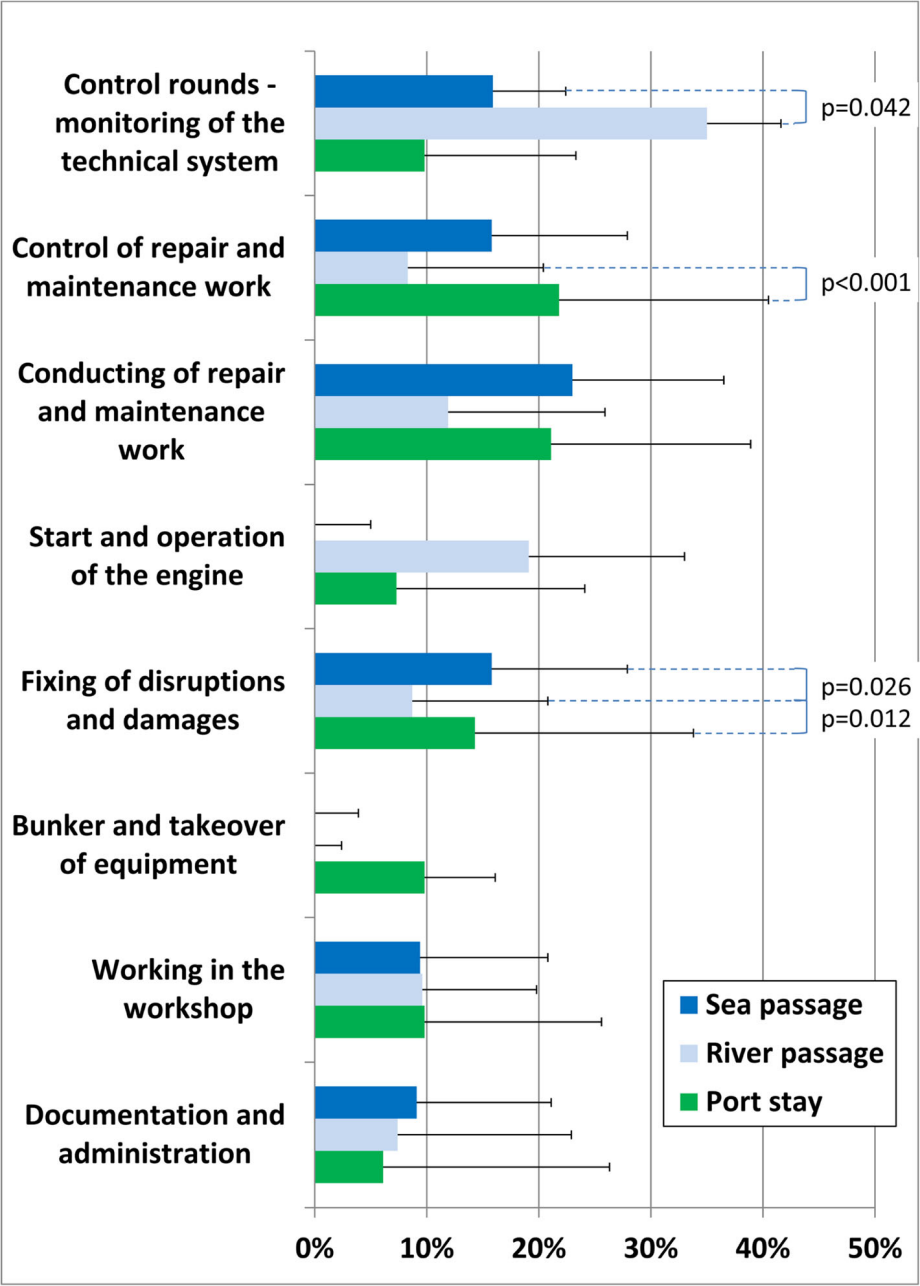
Activity profile for technical officers. p test only in cases of significant differences between the voyage stages (each higher than 0%)

**Fig. 4:**
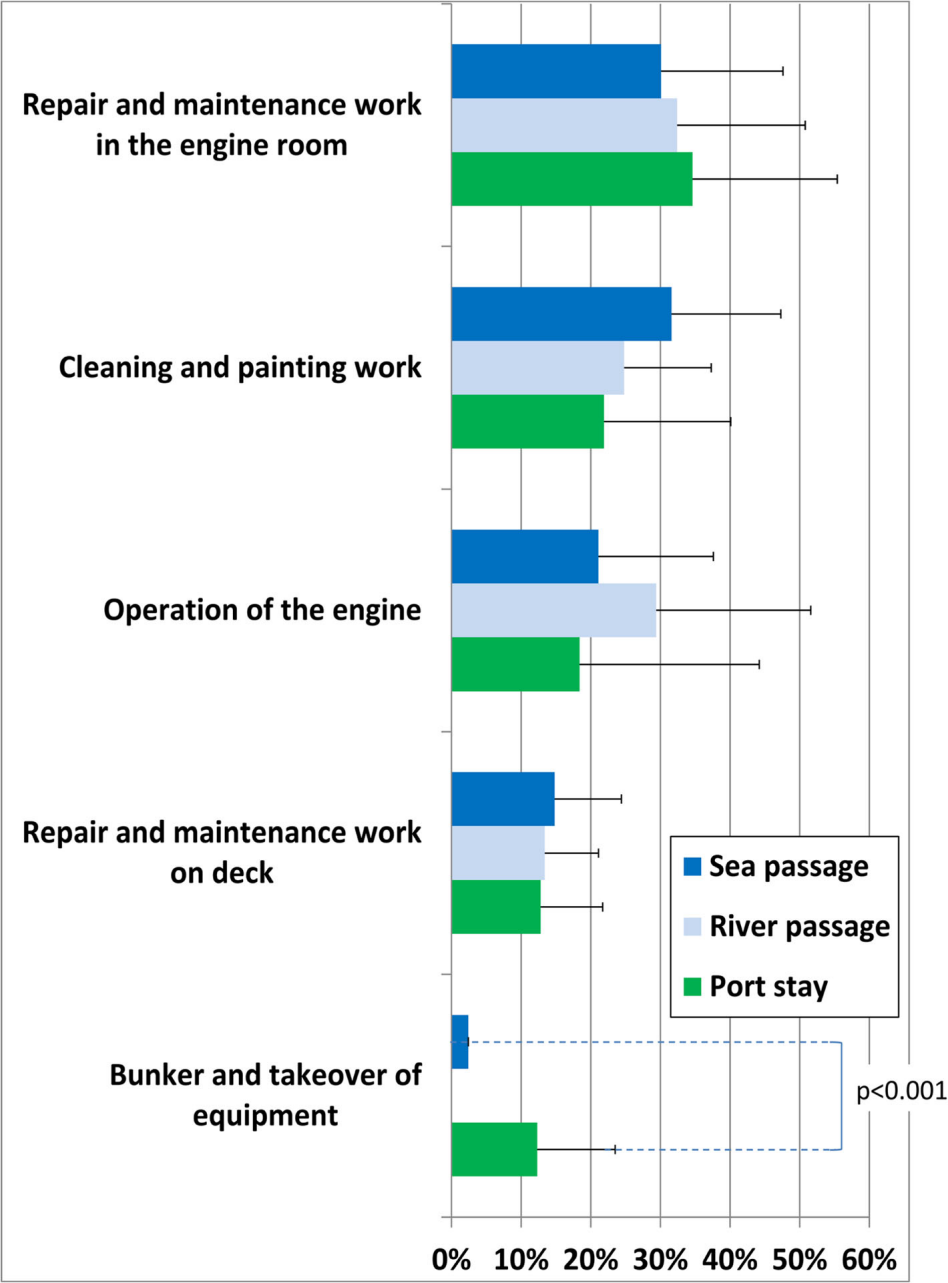
Activity profile for engine room ratings. p test only in cases of significant differences between the voyage stages (each higher than 0%)

The answers about the activities during the past voyage stage were presented as average percentage with standard deviation (SD). In those cases, in which the percentage values in two voyage stages were higher than 0%, the statistical significance of these differences was calculated using the Mann-Whitney U test. Significant findings were presented in the Figs. 1, 2, 3 and 4. All indicated *p*-values were two-sided, and a p-value of < 0.05 was considered to be statistically significant.

## Results

No seafarer entered any additional task in the given job activity list. Figures 1, 2, 3 to 4 summarize the average percentage of active time spent on the various essential activities on board, differentiated according to the above mentioned four occupational groups. As no significant differences (*p* > 0.05) were observed in the activity profile on board of feeder ships or large container ships (with the exception of loading and unloading in port; *p* = 0.045), the data were merged together.

### Nautical officers

As illustrated in Fig. 1, nautical officers cover a very diverse range of activities, which differs during the voyage stages. During port stay, the main focus of activity is on the preparation and monitoring of the loading process, on control rounds on deck as well as on administrative and documentation tasks. The calculation of vessel stability during container loading, equipment control and bureaucratic administration, especially in dealing with port authorities (for example, port state control, customs, immigration and port health authorities) occupy a larger proportion of nautical officers’ working time during port stay.

During the river and sea passage, more than 50% of the working time consists of monitoring / watching of the navigation area and about 10% of navigation. Furthermore, these voyage stages are used, to a greater extent, for documentation and administration work. During the river passage, the supervision of the mooring and unmooring as well as arrangements with the pilot take up additional working time.

### Deck ratings

There are also differences between voyage stages in the job activity profile for deck ratings. In port, the main tasks are (preparation and follow-up) activities for loading and unloading the vessel. In addition, security and watchkeeping tasks are required to a considerable extent (Fig. 2). During the other voyage stages, cleaning, painting and maintenance work predominates on the ship.

During the river passage, the deck ratings are assigned as helmsmen (to operate the rudder according to the pilot/captain’s instructions) and to assist the pilot in the transfer. Furthermore, in this voyage stage and especially during sea passage, more repair and maintenance work is conducted on deck. Above all, the sea passage is often used to carry out cleaning, painting and maintenance work on the ship.

### Technical officers

In comparison to nautical officers, the profile of the technical officers less often differs significantly between the various voyage stages. Their tasks include control, repairs and maintenance throughout the entire voyage. The bunkering of fuel and the takeover of equipment represent important tasks for the technical officers during stays in port.

The start and operation of the ship’s engine (including all controls) takes a long time both during port stay and during river passage (Fig. 3).

### Engine room ratings

The average working day of the engine room ratings is characterized by a high degree of routine with a high proportion of repair and maintenance activities in the engine room and on deck. Except for a slightly larger proportion of cleaning and painting work during sea passages, no noteworthy differences were observed in the activity profile between the various voyage stages (Fig. 4).

## Discussion

Developing job descriptions and planning seafarers’ work schedules necessitate good knowledge of the shipboard activities. The fact that none of the 323 participants surveyed entered any further relevant activities concerning their recent phase of voyage episode indicates that the activity profile list provided according to the previously conducted interviews and job descriptions is complete. When selecting the phase of voyage to be evaluated, the examiners emphasized that a period with a normal workload was needed in order to determine the activities of a routine operation. Certainly, there are also phases with exceptional workloads on board. However, the research focus of this study was not on those phases.

The approach of this study from the perspective of occupational science has shown that nautical officers and deck ratings have a higher variety of activities which differ between voyage stages. In comparison, the activities of the employees in the engine room varied less, which suggests a higher degree of routine tasks for these professional groups. Disturbances or irregularities of the ship’s engine naturally make immediate alterations to their job activities necessary.

The activities of all four occupational groups varied most during port stay and less during the sea passage. This has already been previously described [12]. This confirms the assumption that the sea passage is characterized by more continuity and routine. The seafaring routine has repeatedly described to be psychophysically demanding [6, 7, 13]. In such stressful workplaces - at least episodically during port stay - the sea passage as the voyage stage with less stress is particularly important for the employees’ recreation and rest. This applies especially to crews on ships in worldwide trade with sea passages often lasting for weeks [14, 15]. However, it has also been described that ship crews have experienced these phases as monotonous and consequently as mental stress [16]. Since the work diversity during the voyage stages is obviously different between the shipboard professional groups, further studies are recommended to explore if stress assessments should take these influences into account.

Supervisory and control tasks are a focal point of job activity for nautical and technical officers [3, 17]. As the officers are only able to perform the most necessary documentation and administrative tasks during the demanding port stay, the sea passage is often used to prepare and follow up these activities.

Depending on the rank of the officer (captain, 1st, 2nd or 3rd nautical officer, if applicable), additional activities (for example, regarding personnel management, stability calculation, navigation planning and health care) exist, which are not differentiated in this study. Since some repair services can only be conducted when the engine is stopped, there is also a somewhat greater amount of repair and maintenance work for engine room personnel during port stay.

While the deck personnel (nautical officers and deck ratings) perform duties on the bridge, on deck, in the loading area and partly also in the port, the engine room personnel stay almost exclusively in the engine department and adjoining workshop (below deck). During port stay, the nautical officers, in particular, have external professional contact with port authorities, shipping companies, charterers and pilots.

It is assumed that the activity profiles on other types of ships (for example, bulk carriers or passenger ships) are different from those on container vessels. This type of ship was chosen in this study as container ships are of great and increasing importance for global trade [10]. In addition, only container ships were selected that are at least temporarily deployed in the North/ Baltic Sea and thus have to complete a faster sequence of ports. It is known that the workload on such ships (so-called feeder vessels) is particularly high, especially due to the lack of rest periods during longer sea passages and the usually more psychophysically demanding port turn-arounds, at the latest after a three-day voyage [7, 15].

Another difference in work requirements can be found between smaller feeder container ships and large container ships, as the latter often use land-based personnel during port stay to relieve the crew of the ship in this voyage stage [14, 18]. Correspondingly, differences in this study between feeder and large container vessels are only with regard to the unloading and loading activities in port. Although these tasks were carried out by dock workers on the four large container ships examined, the relief achieved here for the on board personnel resulted in an increase in other work requirements during stays in port.

As a limitation of this study, inactive time or breaks of the seafarers were not recorded. Thus, it is not possible to demonstrate the actual distribution of time allocated to different tasks during the day. The findings of this study confirm the assumption that the activities during the sea passage varied less, which may probably lead to a lower stress level compared to port stay or the river passage. However, due to the fact that the stress load of the activities in the different voyage stages was not weighted or objectified, a job risk-assessment is not possible on the basis of this study. Furthermore, work intensity cannot be derived from this study as the various job activities require different degrees of effort. This was, however, not an objective of the present study and should be focused on in future field studies on board, taking into account the knowledge gained here.

## Conclusions

This study demonstrates the distribution and differences of the tasks of the shipboard crews during the various voyage stages. The activities of all four occupational groups varied most during port stay and less during the sea passage. It could be assumed that the high diversity between the voyage stages may indicate stronger job-related stress, but the study design used is not suitable for analysing or confirming this hypothesis. To prepare maritime trainees for the expected job-related requirements and to identify the most suitable opportunities for refreshing during a voyage, future maritime studies about stress levels should take the differences in the activity profile between occupational groups and the voyage stages into account.

## Abbreviations

ISPS: International Ship and Port Facility Security Code
SD: Standard Deviation
